# Developing and Implementing an Integrated Family Education Program (IPMD‐F) for Parents of Children With Reading Difficulties: Insights From an Action Research Study

**DOI:** 10.1002/brb3.70269

**Published:** 2025-01-29

**Authors:** Hülya Tercan, Pınar Bayhan

**Affiliations:** ^1^ Faculty of Health Sciences, Child Development Department Hacettepe University Ankara Turkey

**Keywords:** action research, dyslexia, family education program, neurodiversity, reading difficulty

## Abstract

**Purpose:**

This research aims to identify the problems and needs of families of children with reading difficulties, develop an Integrated Process‐Based Family Education Program (IPMD‐F) to address these needs, and implement it.

**Methods:**

The study used a community‐based participatory action research approach, following a four‐stage process: general information collection, needs identification and action plan creation, development and implementation of the IPMD‐F, and evaluation. Conducted during the 2023–2024 academic year in Ankara, Turkey, with 16 volunteer parents of children diagnosed with learning disabilities, data were collected using qualitative and quantitative tools. Qualitative data were analyzed using thematic reflexive analysis, while quantitative data were analyzed using the Wilcoxon signed‐rank test to determine pre‐test and post‐test score differences.

**Finding:**

Findings derived from a synthesis of personal development theory, a holistic approach, and a neurodiversity perspective revealed that the program assisted parents in attaining a holistic outlook, reinforcing parent–child communication, and cultivating coping strategies. Our assessment also substantiated the program's efficacy in practice.

**Conclusion:**

This study highlights that the IPMD‐F can be a significant resource for families of children with reading difficulties and suggests broader implementation of such programs.

## Graphical abstract

This figure illustrates the development and implementation of the Integrated Process‐Based Family Education Program (IPMD‐F) for parents of children with reading difficulties. The IPMD‐F was designed based on a systematic needs assessment and followed a structured process of development, implementation, and evaluation. The program aims to enhance parents' understanding and management of reading difficulties in their children through a holistic and participatory approach. The process involved initial data collection, identification of needs, creation of an action plan, and final program evaluation, all of which contributed to the program's effectiveness in supporting families and improving parent–child communication.

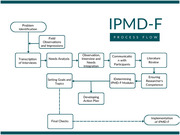

## 1 Introduction

In the clinical field, researchers classify childhood developmental disorders into different diagnostic categories using the *Diagnostic and Statistical Manual of Mental Disorders* (DSM V) published by the American Psychiatric Association (APA)(2013). In the DSM‐V, specific learning disorders are categorized by academic skills that are significantly below the expected level for the individual's chronological age and cannot be explained by neurological, visual, or auditory conditions. Reading difficulties, which fall under the umbrella of learning disabilities and various neurodevelopmental disorders, are characterized by deficits in reading accuracy, fluency, and/or comprehension.

The underlying causes of reading difficulties are diverse and multifaceted. Dyslexia, one of the most common reading difficulties, is known to have a complex etiology that includes genetic, cognitive, neurobiological, and environmental factors (Ozernov‐Palchik et al. 2016). Classified as a neurodevelopmental disorder by the DSM‐V, dyslexia is characterized by significant difficulties in reading, writing, and spelling skills (Sanfilippo et al. 2020), and it is known to affect approximately 5%–10% of children (Elliott and Resing 2015; Shaywitz and Shaywitz 2003). From the early stages of literacy development, the reading process is considered the strongest predictor of academic performance, independent of children's social skills (Martínez, Maurits, and Maassen 2023). Children with reading difficulties may experience significant declines in academic performance, which can adversely affect their social interactions, overall psychological well‐being, and family relationships (Krämer, Möller, and Zimmermann 2021).

The extant literature indicates that children with reading difficulties tend to encounter greater academic challenges than their peers, resulting in lower‐than‐expected academic performance in later years (Volkmer, Galuschka, and Schulte‐Körne 2019). These children's inclusion in suitable educational and assistance programs at an early age can facilitate the remediation of their reading deficiencies, augment their academic performance, and diminish the likelihood of their social isolation (Heady et al. 2022; Sanfilippo et al. 2020).

The roles of parents and teachers are of paramount importance in this process. The home and school environments represent the two most significant settings that can directly influence a child's cognitive, social, and emotional development (Ma et al. 2016). The ramifications of reading difficulties are not limited to the individual; they also affect family dynamics. Families frequently experience elevated stress levels, feelings of helplessness, and a lack of resources to adequately support their children (Abd Rauf et al. 2021). Today, many researchers recognize the important role that a strong positive connection between home and school plays in children's development and education (Ma et al. 2016; van Bergen et al. 2017). Recent research has shown that family–school collaboration plays a significant role in enhancing children's academic achievement (Forteza‐Forteza et al. 2021; Gerdes et al. 2022; Villiger 2021). The collaboration between families and educators is one of the most important factors in ensuring that children receive more effective support in their educational process (Grigorenko et al. 2020).

The foundation of family education lies in the idea of strengthening family communication and interaction by supporting family members (Graham Özyürek and Şahin 2015). Therefore, family education programs are an effective tool for parents to learn how to contribute to their children's education (Abror Huda and Yayah Haenilah 2024). These programs play a crucial role in developing parenting skills that enable parents to more effectively address their children's reading processes and the issues arising from reading difficulties (Leslie et al. 2024; Torppa et al. 2022). Current literature emphasizes the need for further research and awareness in this field and suggests that educational policies should be developed accordingly (Abed and Shackelford 2023; Ma et al. 2016).

It is evident that reading difficulties have the potential to pose considerable challenges, affecting children in various domains, including their academic achievements, social interactions, and overall psychological well‐being. These issues frequently spill over to affect families, resulting in heightened stress levels and the necessity for effective strategies to support their children's learning processes (Dunbar 2015; Grigorenko et al. 2020). Despite the existence of various educational interventions aimed at families (George, Kidd, and Brack 2011), there are significant gaps in the literature regarding the holistic needs of these families through structured programs and addressing the children's conditions from a neurodiversity perspective. The principal objective of this family‐based intervention study is to meticulously identify the challenges encountered by families of children with reading difficulties and to construct the (IPMD‐F) to address these needs. This program was structured to practically respond to the identified needs of families and ensure effectiveness by adopting a participatory approach. The IPMD‐F program was designed to equip parents with the necessary tools and strategies to effectively support their children, fostering holistic perspectives and empowerment. This study was guided by several key questions: (a) What are the primary educational needs and challenges faced by families of children with reading difficulties? (b) How do parents perceive and experience their children's reading difficulties? (c) What resources and support systems do families utilize, and what barriers do they encounter in accessing these resources?

Given our research questions and an extensive review of the literature on the educational needs of families of children with reading difficulties, as well as the necessity for robust strategies to address these needs (Ma et al. 2016; Grigorenko et al. 2020; Torppa et al. 2022), we formulated the following hypotheses: (a) families of children with reading difficulties have significant unmet educational needs; (b) the implementation of targeted family education programs can facilitate the development of effective communication strategies and coping mechanisms; (c) an integrated process‐based approach has the potential to markedly enhance the overall well‐being and problem‐solving abilities of these families.

## 2 Materials and Methods

### 2.1 Research Design

**FIGURE 1:**
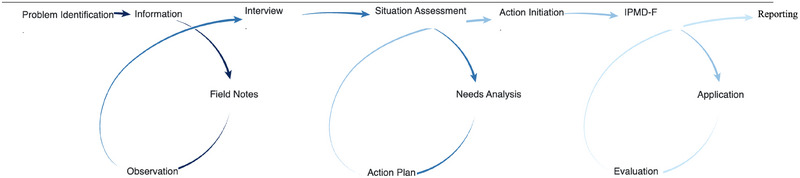
Participatory action research cycle of the study. IPMD‐F: Integrated Process‐Based Family Education Program.

This study employs a participatory action research approach, a subtype of action research that involves collecting and analyzing data to take action and facilitate change or transformation within a practical context (De Oliveira 2023; Cornish et al. 2023). In essence, it represents a distinct philosophical perspective on social inquiry to engender social transformation (Kemmis, McTaggart, and Nixon 2014). In this study, we adopted the semi‐structured interview technique due to its reliability and effectiveness in gaining an in‐depth understanding of participant perspectives, as well as its flexibility and open‐ended structure. We also adopted reflexive thematic analysis (Braun and Clarke 2013) to gain immediate insights from the participant narratives and to facilitate the conceptualization of categories, themes, and concepts. Besides, the Standards for Reporting Qualitative Research (SRQR) were adhered to in the qualitative research component of the study. As demonstrated in Figure 1, the design of this study also incorporated sequential action research cycles at each stage.

### 2.2 Theoretical and Philosophical Background of the Program

The theoretical foundations of the IPMD‐F are based on theories grounded in various disciplines. The first theoretical approach is Bronfenbrenner's Ecological Systems Theory (Bronfenbrenner 1975). In developing the IPMD‐F program, a holistic approach was taken to address reading difficulties through the interaction of the child with family, school, and other environmental factors. Another theoretical foundation of the program is Epstein's Family Involvement Theory, which posits that active family involvement in a child's education positively impacts the child's academic success (Epstein 1995). The program's final thematic structures are conceptualized using Carl Rogers' Humanistic Psychology Theory, specifically the Personal Empowerment Theory (Rogers 1961). This theory aligns with the IPMD‐F's goal of enabling individuals to bring about change and transformation in their lives by utilizing their internal resources.

The most important philosophical underpinning of IPMD‐F is holism, a comprehensive approach addressing life's complexities and diversity (Jeder 2013). This holistic perspective, influential in health and education, considers the child and family within their environmental contexts, addressing needs and expectations comprehensively. Moreover, the IPMD‐F is based on the neurodiversity approach, a recent advancement in dyslexia and reading difficulties. Initially emerging in the 1990s in the context of autism, neurodiversity now applies to conditions such as dyslexia, dyspraxia, dyscalculia, ADHD, and Asperger syndrome (Armstrong 2010). From this perspective, IPMD‐F collaborates with families to support children's differences, offering an innovative approach aligned with neurodiversity principles.

### 2.3 Participants

All procedures performed in studies involving human participants were in accordance with the ethical standards of the institutional and/or national research committee and with the 1964 Helsinki Declaration and its later amendments or comparable ethical standards. Research Ethics Committee granted the relevant approval for our study (Number: 16969557‐903, Decision Number: 2022/08‐26, and Date: February 15, 2022). The study was conducted during the 2023–2024 academic year at a private special education and rehabilitation center in Ankara, the capital of Turkey. It involved the parents (14 mothers and 2 fathers) of 16 children who met the DSM‐V (APA 2013) criteria for specific learning disorder and were diagnosed with specific learning difficulties by a child psychiatrist, as documented in a health committee report. These children were receiving education at the special education center. The criterion sampling method was used to select the study group. The selection was based on the parents' willingness to participate, and their consent was obtained through an informed consent form. The demographic characteristics of the parents who participated in the study are presented in Table 2.

### 2.4 Instruments

Both qualitative and quantitative data collection tools were utilized to obtain the data. The data for this study were collected through a four‐stage structure consisting of interconnected action research cycles, with specific data collection instruments designed for each stage.

#### 2.4.1 Family and Child Characteristics Assessment Form

In the first stage of the research, detailed information about families and children was gathered using the “Family and Child Characteristics Assessment Form.” The questions were developed based on a literature review and reviewed by two doctoral experts in assessment and evaluation, as well as an independent researcher. The final questionnaire was administered to participating parents.

#### 2.4.2 Semi‐Structured Interview Form

Prior to the educational program, face‐to‐face interviews were conducted using a semi‐structured interview technique. The interview form was developed following a literature review on children with reading difficulties and their families. A pool of relevant questions was created, reviewed by two academics specializing in qualitative and action research, and four experts including a special educator and a child development specialist. The final form, comprising 8 main questions and 15 supplementary questions, was used in the initial family interviews.

#### 2.4.3 Parent–Child Communication Scale

The “Parent–Child Communication Scale” (PCCS) by Kahraman (2016) was used to measure the quality of communication between parents and children as both a pre‐test and post‐test. The scale includes 27 items across five dimensions: “openness to sharing,” “barrier‐free listening,” “respect‐acceptance,” “sensitivity,” and “problem‐solving.” Evaluated on a five‐point Likert scale, higher scores indicate better communication. Validity studies reported a KMO coefficient of 0.910 and Bartlett's Sphericity test *χ* ^2^ value of 5335.151 (*p* < 0.001). Reliability analyses showed a test–retest correlation of *r* = 0.899.

#### 2.4.4 Focus Group Interview Form

At the program's end, focus group interviews were conducted to understand families' experiences and perceptions. The semi‐structured format allowed for flexible discussion, facilitating the expression of families' thoughts and emotions regarding their participation in the program.

### 2.5 Procedures

The present study was conducted in four stages, following the participatory action research cycle shown in Figure 1. In the first stage, basic information about the families who voluntarily agreed to participate in the study was collected, and a detailed literature review was conducted on the subject. In the second stage, face‐to‐face interviews were conducted to assess the current situation and identify the needs from the perspective of the families regarding reading difficulties. In the third stage, based on the current situation and needs identified in the previous stage, an action plan was prepared, and the Integrated Process‐Based Family Education Program (IPMD‐F) was developed and implemented for parents over an 8‐week period. In the final stage, the program and process evaluation was conducted through focus group interviews with the participants, and the evaluations were reported by the researcher.

### 2.6 Development of the IPMD‐F

**FIGURE 2:**
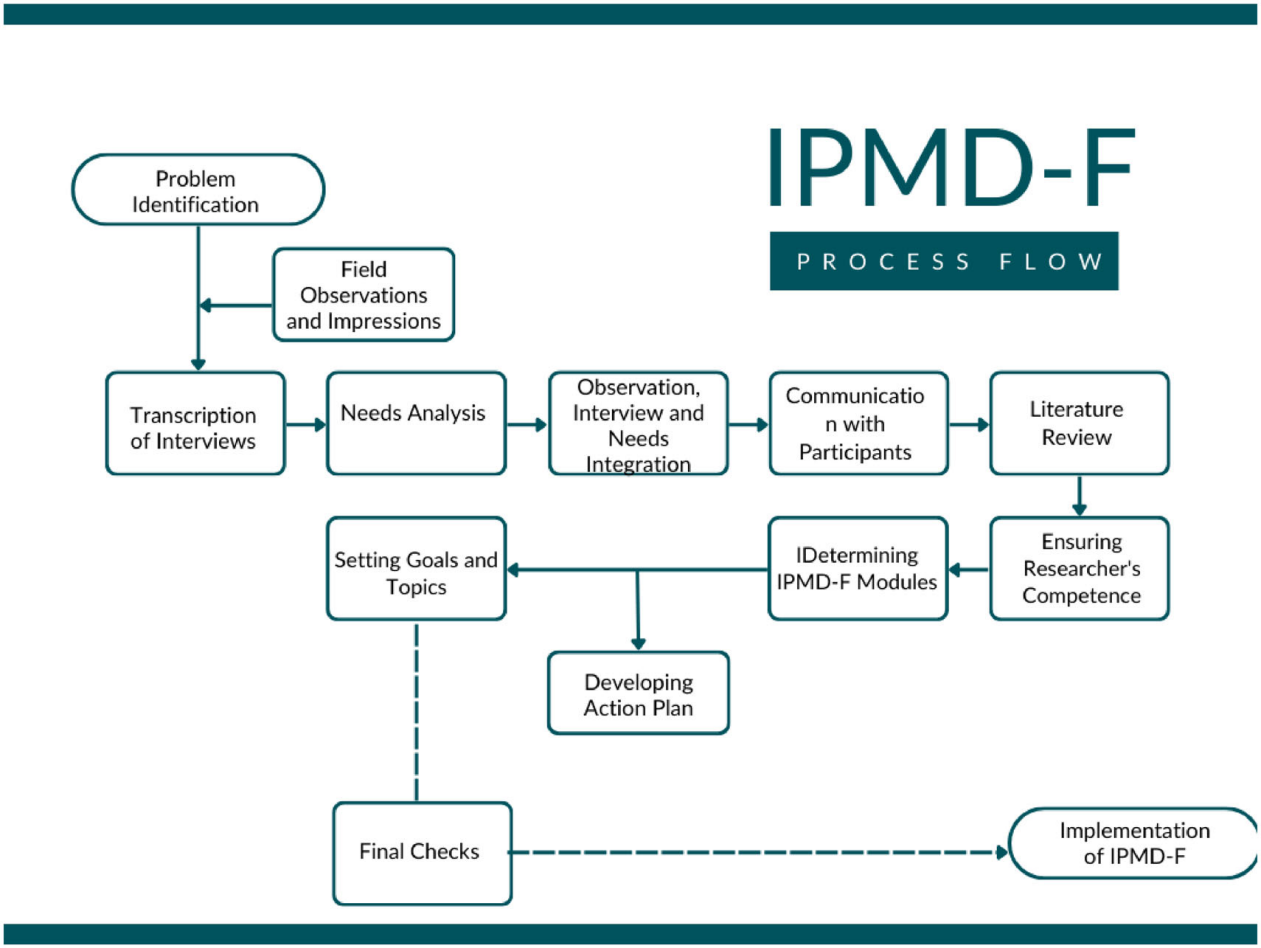
Process flow chart for the development of the IPMD‐F program.

This study, in alignment with the action research approach, consists of a process in which participants actively engage in understanding their situations, identifying problems, and generating solutions. These steps reflect an action research process that focuses on developing the IPMD‐F program in a participant‐centered and needs‐based manner. Each stage involves incorporating participants into the process, obtaining their feedback, and continuously improving the program based on this feedback through a flexible and iterative approach. Initial family interviews, field observations, situation assessments, and needs analyses revealed that the identified problems and their sub‐problems needed resolution. The process flow chart of the action plan developed to address the identified problems and improve the current situation is shown in Figure 2.

### 2.7 Goals and Sessions of the IPMD‐F Program

This family education program is a multifaceted model that addresses the problems and needs experienced by children with reading difficulties and their families in a holistic manner, and it evaluates learning difficulties comprehensively. The educational sessions were conducted according to a specific plan. The target group, duration, and weekly topics of the program are presented in Table 1.

**TABLE 1:** Goals and sessions of the integrated Process‐Based Family Education Program (IPMD‐F).

Target group: families of 7–10‐year‐old children diagnosed with specific learning disabilities and experiencing reading difficulties General objective: to provide integrated support to families of children with reading difficulties Duration: 8 weeks							
Implementation weeks	**Objective**	**Topic**	**Methods and techniques**	**Materials**	**Duration**	**Date**	**Number of participants**
Week 1	– Introduce and communicate with families – Review families' expectations and needs – Provide information about the program – Explain the process and implementation of the program	Introduction and Getting Acquainted	– Welcome meeting – Name–sharing games – Creating and sharing a small family map – Sharing needs analyses – Small group activities	– Note papers – Colored pencils – Projector – Table and chairs – Flipchart	60 min	…/….	16
Week 2	– Provide information about reading difficulties – Discuss characteristics of children with reading difficulties – Share families' definitions and experiences of their children's reading difficulties	Understanding Reading Difficulties	**–** Presentation on learning difficulties and reading difficulties – Case study presentations – Reading difficulty simulation – Group discussions	– Note papers – Projector – Table and chairs – Flipchart	60 min		16
Week 3	– Learn positive communication techniques with children – Develop motivation strategies – Learn positive reinforcement techniques	Motivation System and Positive Communication Techniques	– Creating a motivation board – Setting goals – Bonding and strengthening activities – Practicing positive expressions – Role‐playing – Sharing positive experiences – Empathy exercises	– Note papers – Cork boards – Projector – Table and chairs – Flipchart	60 min		16
Week 4	– Introduction to the integrated process model developed for families of children with reading difficulties – Learn details about the integrated process‐based model	Understanding IPMD‐F	– Introduction to IPMD‐F – Holistic and neurodiversity approach – Identifying learning styles	– Note papers – Projector – Table and chairs – Flipchart	60 min		16
Week 5	– Learn strategies and methods for holistic support **–** Provide suggestions related to integrated processes – Develop strategies to support these processes	Strategy Development for Holistic Support	– Summary and review presentation of IPMD‐F – Understanding holistic support mechanisms – Problem identification and solving exercises – Time management practices – Group discussions and experience sharing	– Note papers – Projector – Table and chairs – Flipchart	60 min		16
Week 6	– Create supportive environments at home – Discuss how to make supportive materials used in the program accessible at home – Create support times and rituals at home	Creating Suitable Environments for IPMD‐F Support	– Analysis of home and family environment – Focusing on home interests – Attention to sensory stimuli – Study and break times – Preparing learning materials – Effective use of technology – Fun learning experiences	– Note papers – Projector – Table and chairs – Flipchart	60 min		16
Week 7	– Evaluate and record the feasibility of techniques used in the program at home – Develop strategies for solving potential problems – Provide information on how families can access support groups and resources	Monitoring Progress and Solving Problems	– Progress journals and tracking – Group discussions and experience sharing	– Note papers – Projector – Table and chairs – Flipchart	60 min		16
Week 8	– Evaluate the overall process of the program – Plan what needs to be done to ensure the sustainability of the program in the future – Plan for future support	Evaluating Results and Future Steps	– Group evaluation sharing (Training journey map activity) – Individual evaluation notes – Survey and feedback forms – Discussing future steps	– Colored papers – Large colored cardboard – Colored pencils – Scissors – Projector – Flipchart	60 min		16

### 2.8 Data Analysis

In this study, the analysis of qualitative data was conducted using Braun and Clarke's (2006, 2013, 2022) thematic analysis approach, which includes the stages of data collection, data preparation, coding, and theme development (Braun et al. 2022). The process involved transcribing the interviews and coding the data based on recurring themes. These data were then reviewed and refined in collaboration with field experts. Quantitative data were downloaded into an Excel spreadsheet and cleaned. Since action research does not require a control group like experimental studies, the Wilcoxon signed‐rank test was conducted to determine whether there was a statistically significant difference between the pre‐test and post‐test quantitative data obtained from the scales and questionnaires. SPSS 23 (IBM SPSS Statistics for Windows, Version 23.0. 2015) software package was used for the analysis.

### 2.9 Reliability of Action Research

In action research, validity and reliability are evaluated from a perspective different from traditional quantitative research. Fundamentally, action research should be “reliable” by being free from biases and simplistic conceptualizations (Kemmis, McTaggart, and Nixon 2014). Various strategies were employed in this study to meet and satisfy verification criteria.

#### 2.9.1 Diversified Data Collection Sources

To enhance reliability, the study utilized semi‐structured interviews, questionnaires, and research diaries. Results from these sources were compared for further reliability.

#### 2.9.2 Participant Validation

Weekly feedback meetings with participating parents were held to discuss themes and verify interpretations and conclusions from the data.

#### 2.9.3 Researcher Triangulation

Thematic analysis results were consistent with the outcomes from two special education teachers and two experts who reviewed the semi‐structured interview forms.

#### 2.9.4 Explanation

The study was conducted in four stages, following participatory action research cycles, with detailed explanations for each stage.

#### 2.9.5 Written Documentation

All individual and focus group interviews conducted using semi‐structured tools were documented in written form.

## 3 Results

The results of this study are presented in accordance with the four stages of the research process: obtaining general information, identifying needs and developing an action plan, implementing the IPMD‐F, and evaluating the program and process in the final stage.

### 3.1 Stage 1: Results Related to General Characteristics

In the first stage, data were collected using the “Family and Child Characteristics Assessment Form.” This tool provided comprehensive demographic, developmental, and historical information about the families and their children. The general characteristics of the participants and their children are presented in Table 2.

**TABLE 2:** General demographic characteristics of the participants and their children.

Characteristics	Frequency	Percentage %
Parent demographics		
Sex Female	**14**	87.5
Male	2	12.5
Age 21–30 years	3	18.75
31–40 years	**9**	56.25
41–50 years	4	25
Education Primary/middle School	3	18.75
High School	3	18.75
Undergraduate	**8**	50
Graduate	2	12.5
Living with spouse	16	100
Employment status Working	6	37.5
Not working	**10**	62.5
Income level Middle	7	43.75
Above middle	**9**	56.25
Number of children One	**8**	50
Two	3	18.75
Three	5	31.25
Child characteristics		
Highly active	**12**	75
Excitable	8	50
Shy and timid	6	37.5
Gender Female	6	37.5
Male	**10**	62.5
Age 7 years	5	31.25
8 years	**6**	37.5
9 years	3	18.75
10 years	2	12.5
Grade First grade	3	18.75
Second grade	**7**	43.75
Third grade	4	25
Fourth grade	1	6.25
Fifth grade	1	6.25
Age of initial symptoms 5 years and under	3	18.75
6 years	5	31.25
7 years	**7**	43.75
8 years	1	6.25
Diagnosis age 7 years and under	4	25
8 years	**10**	62.5
9 years	2	12.5
Special education (receiving)	16	100
Special education start age 7 years and under	5	31.25
8 years	**9**	56.25
9 years	1	6.25
10 years	1	6.25
Regular medication use Yes	8	50
No	8	50
Own room Yes	**13**	81.25
No	3	18.75
Study area Yes	**15**	93.75
No	1	6.25
Sufficient educational materials Yes	**9**	56.25
No	7	43.75

*Note*: The bolded items refers to critical demographic characteristics and key information regarding the participants and their children.

### 3.2 Stage 2: Results of Situation Assessment and Needs Identification

In the second stage, individual interviews were conducted with parents using a semi‐structured interview form. The interviews thoroughly examined topics such as the diagnosis of children's reading difficulties, the effects of this diagnosis, and family communication and attitudes. Through thematic analysis of the data collection tools from the initial interviews, general categories emerging from the data of all participants were identified. Following the identification of general categories, the main themes and sub‐themes of family interviews were formed under common categories. Based on the interviews conducted with the participants, the general categories emerging from the initial interviews were conceptualized as “diagnostic process, effects of reading difficulties, family communication and attitudes, difficulties, needs, education system and expectations, government and community support.” After re‐evaluating the findings of the initial interviews, the general categories were organized into common categories, and main themes and sub‐themes were identified for each common category. The main themes and sub‐themes obtained from the initial individual interviews with parents are presented in the section on initial thematic structures in Table 3.

**TABLE 3:** Initial and final thematic structures of the participants.

Common categories	Initial thematic structures			Final thematic structures		
Diagnostic process	**Main themes**	**Sub‐themes**	***f***	**Main themes**	**Sub‐themes**	***f***
	1.1 Perceived differences	Attributing to a cause	12	Gaining knowledge and awareness	Retrospective awareness	12
		Retrospective awareness	14		Understanding causes and effects	13
	1.2 Awareness	Coping with being different	16		Making sense of the process	14
		Child's intense and recurrent experience of distress	14		Acceptance	12
	1.3 Difficulties and barriers	Lack of information	13	Empathy and understanding	Changing perception of reading difficulty	15
		Elderly family members and elose environment	10		Understanding and sharing child's experiences	13
		Systemic difficulties	10		Empathic bonding with other families	16
Effects of reading difficulty	2.1. Psychological effects	Feeling sad about child's condition	16	Confidence and optimism Mood changes Developing coping skills Self‐confidence	Realizing not being alone	15
		Guilt and helplessness	13		Relief feeling	14
		Parental labeling	12		Hope for the future	12
		Loneliness	12			
		Feelings of failure and inadequacy	11		Ways to overcome difficulties	13
		Loss of motivation and self‐confidence	10		Feeling understood	14
					Gaining self‐confidence	11
		Reluctance to seek help	11		Trusting and believing in child's potential	12
	2.3. Impact on daily life	Changes in life routine	10	Balancing	Finding time for oneself	11
		Lack of time	11		Managing time	12
	2.4. Impact on educational life	Rigid teaching approaches	12	Increased participation in education Active communication with teachers Understanding and applying Holistic processes Adaptation	Gaining confidence and motivation	12
		Child's labeling	14			
		Negative perceptions of peers	12			
		Balancing and catching up efforts	12		Developing a holistic perspective	15
		Conflict with the teacher	14		Adapting home environment	11
		Lack of progress, lack of results	14		Balancing expectations	13
					Focusing more on child's strengths	14
		Repeated failure cycle	16			
	2.5. Impact on family relations	Different parenting experience	13	Parental Empowerment Taking joint responsibility Developing positive Communication reflection	Changing parenting attitudes and roles	15
		Parental stress	14			
		Conflict between spouses	9		Developing positive communication with child	14
		Communication problems with child	14		Reflections of well‐being	16
		Increased responsibility	11			
		Communication problems within the family	10			
		Challenging motherhood role	13			

### 3.3 Stage 3: Implementation of IPMD‐F as an Action Plan

**FIGURE 3:**
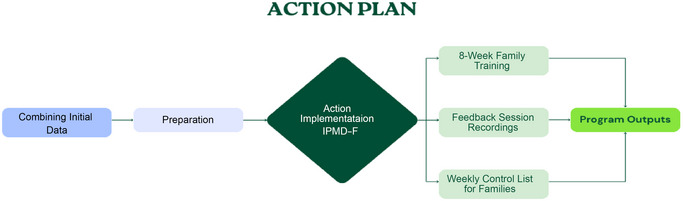
Implementation of the action plan.

In this stage, considering all the findings obtained from the first two stages, the action plan was created and implemented. The implementation of the action plan was carried out as shown in Figure 3.

At the conclusion of the IPMD‐F action research, a comprehensive qualitative analysis was presented, detailing the real‐time progression of the research and the emergent themes related to participants' transformational learning experiences. The transformational thematic structures of the families are shown in Table 3.

### 3.4 Stage 4: Results of Program and Process Evaluation

This stage is the final stage in which the IPMD‐F program, developed as an action plan, and the entire action research process are evaluated. In this final stage, a focus group discussion was conducted with parents to assess the effectiveness of the program.

### 3.5 Results of Focus Group Interview

The focus group interview conducted during the evaluation phase centered on the families' experiences, feelings, and thoughts regarding their participation in the education program process and activities. The responses provided by the families during the focus group interview were evaluated and thematically analyzed. The findings from the thematic analysis were then combined with the initial individual interview data to create a consolidated table (Table 3) showing the participants' initial and final thematic structures. The primary purpose of evaluating the initial and final thematic structures together is to examine the thematic structures displayed by the participants at the beginning and end of the action plan, thereby revealing the developments and changes that occurred throughout the process. The initial and final thematic structures of the participants are presented in Table 3.

The underlying purpose of the joint analysis of the emerging themes is to initially discuss and evaluate the existing schemas and related issues of the participating parents, reflect these in the education program, and subsequently, at the end of the program, enable the parents to reflect on the program and evaluate their new schemas. As seen in Table 3, the findings from the thematic analysis obtained at the end of the education program indicate that there has been “development and change” in the thematic structures of the participants in the categories of “diagnostic process” and “effects of reading difficulties.”

The initial and final thematic structures in Table 3 demonstrate the transformation in the participants' perceptions and cognitive processes related to their experiences with children who have reading difficulties.

### 3.6 Parent–Child Communication Scale Pre‐Test and Post‐Test Results

The result of the Wilcoxon signed‐rank test showed that participants’ pre‐test and post‐test PCCS scores significantly differed (*z* = 2.028, *p* < 0.05). This finding suggests significant, positive changes in the communication between parents and their parents participating in the IPMD‐F program in all five components of PCCS, documenting the efficiency of the program in enhancing parent–child communication (Table 4).

**TABLE 4:** Wilcoxon test results of participants' PCCS pre‐test and post‐test.

		*N*	Mean rank	Sum of ranks	*Z*	*p*
	Negative ranks	0	0.00	00	2.028^a^	0.043
Pre‐test	Positive ranks	15	4.33	26.00		
Post‐test	Equal	1	2	2		
	Total	16				

*Note: p*‐value: below the significance level of 0.05; *z*‐value: obtained through the Wilcoxon signed‐rank test and calculated based on positive ranks.

^a^ Based on the positive ranks.

## 4 Discussion

In accordance with the participatory action research model, this study identified numerous support needs in psychological, financial, educational, and familial areas based on the analyses conducted in the first two phases and the information gathered from the parents' experiences. A holistic approach from a neurodiversity perspective was adopted in developing the action plan, considering these identified needs. The qualitative data obtained were analyzed using Braun and Clarke's (2006, 2022) reflexive thematic analysis approach (Braun et al. 2022). In the literature, reflexive thematic analysis is defined as a method that enables researchers to develop an in‐depth understanding of participants' experiences and is known for its flexibility (Nowell et al. 2017; Terry et al. 2017). This method allows researchers to interact with the data, make sense of emerging themes, and better explain the complex processes experienced by participants (Braun and Clarke 2019). In addition, the educational program's emphasis on the neurodiversity perspective provides an important framework for understanding individuals' neurological differences and the impact of these differences on their learning processes (Armstrong 2010; Kapp et al. 2013). The findings, in line with existing literature, emphasize the importance of individualized interventions to meet the support needs of children with reading difficulties and dyslexia and their families (Al Otaiba, Rouse, and Baker 2018; Hulme and Snowling 2016).

In the first phase of the research, which involved gathering information about the families, a substantial amount of fundamental data about the participants and children exhibiting reading difficulties was collected. When compared with existing studies in the literature, the majority of these findings align with the expected outcomes (Hulme and Snowling 2016; van Bergen et al. 2011).

The thematic analysis of the interviews conducted with participants in the second phase of the research revealed that the participants' views were categorized into three main themes: “diagnosis process,” “effects of reading difficulties,” and “needs and expectations.” These themes were further broken down into distinct sub‐themes. The primary themes related to the diagnosis process were conceptualized as “perceived differences, awareness, challenges, and barriers.” Recent studies have focused on the challenges and barriers encountered during the diagnosis process, particularly for individuals with reading difficulties. It has been noted that perceived differences and awareness issues between parents and educators can significantly impact early detection (Catts et al. 2016; Tunmer and Hoover 2019). The second common category, the “effects of reading difficulties,” highlighted “psychological, financial impacts, impacts on daily life, educational life, school environment, and family relationships.” The psychological impacts of reading difficulties, in particular, have been extensively examined in the literature (Elgendi et al. 2021; Francis et al. 2019). Among children with reading difficulties, psychological effects such as anxiety and low self‐esteem are commonly reported (Zuppardo et al. 2023). The financial burdens faced by families, particularly the costs associated with private tutoring and educational resources, are among the significant concerns (Pickard 2024). The findings of this study expand on the literature by emphasizing the widespread impact of reading difficulties on daily life, educational experiences, and family relationships, highlighting the extensive effects on various aspects of a child's life.

All the aforementioned second stage results constitute the most significant justification for addressing the family education program developed in this study from a holistic perspective and through the lens of personal empowerment. It is evident that traditional teaching methods may be insufficient for children with reading difficulties, and it is crucial to adopt approaches that cater to their unique learning needs (Leitão et al. 2017; Ward‐Lonergan and Duthie 2018). The needs assessment and situational analysis stage of this study has shown that the challenges faced by children in their educational lives and school environments can have impacts not only academically but also emotionally, psychosocially, and within the family. Therefore, designing and implementing the developed family education program with a holistic approach distinguishes this study from other family education programs.

The IPMD‐F, developed based on in‐depth parental experiences and needs, aims to alleviate the diagnosis‐related challenges and provide parents with more information and support throughout the process, relying on the real‐time course of the action research and the participants' transformative learning experiences. The thematic analysis findings at the end of the training program revealed that participants experienced “development and change” in their thematic structures in the categories of “diagnosis process” and “effects of reading difficulties.” To illustrate, while initially experiencing profound uncertainty and worry regarding their children's reading disability diagnosis, the majority of participating parents acquired a more nuanced understanding of the process and developed a more supportive approach by the conclusion of the training program. This finding supports the previous research that emphasizes the importance of raising awareness and addressing the educational needs of parents of children with reading difficulties (Giménez et al. 2017; Mudzielwana 2014).

In this study, parents were encouraged to consider reading difficulties as a potential indicator of broader developmental issues, rather than solely an academic concern. For instance, while some parents initially asserted that they did not perceive a direct correlation between their children's reading difficulties and their self‐confidence, they subsequently became more attuned to the significance of providing comprehensive support for their children, encompassing academic, social, and emotional needs. This shift in perspective led them to develop strategies to foster self‐confidence in their children. This holistic approach enabled parents to integrate strategies for coping with reading difficulties not only into their children's learning processes but also into all areas of their development and daily practices. These findings, conceptualized in the context of self‐empowerment theory (Lord and Hutchison 1993; Perkins and Zimmerman 1995; Rogers 1961), demonstrated that parents of children with reading difficulties were able to develop a holistic perspective throughout the program, enhance parent–child communication, and cultivate coping strategies during this challenging journey. For instance, parents began to utilize a more open and supportive language in their interactions with their children, which had a favorable impact on their emotional well‐being. It is anticipated that these changes will not only reinforce the parent–child relationship but also equip children with the capacity to more effectively cope with the challenges associated with reading difficulties.

In addition, the thematic analysis of the focus group interviews following the program yielded findings that were consistent with previous research regarding the critical role of parents in fostering their children's self‐confidence and ‐esteem. Leitão et al. (2017)bib1 underscored the pivotal role of parents in fostering children's self‐perception and ‐confidence, which directly influences their motivation to learn and their social interactions. Similarly, Singer (2008) discovered that parental support in academic settings markedly enhances children's resilience and capacity to overcome learning difficulties. In addition, Terras, Thompson, and Minnis (2009) emphasized the significance of parental involvement in developing children's self‐esteem, asserting that children with consistent family support tend to exhibit enhanced emotional well‐being and academic performance. Overall, this study builds upon previous research by delving deeply into the themes of “increasing self‐confidence and motivation,” “developing a holistic perspective,” “focusing on the child's strengths,” and “balancing expectations” in the context of the family training program. The action plan also underscores the significance of thematic transformations within families (see Table 2) and highlights an enhanced parental involvement in education, more active parent–teacher communication, and the cultivation of comprehension and adaptation to holistic processes and pivotal skills pertaining to the child's educational trajectory. Therefore, our results demonstrated that the IPMD‐F program is not only aligned with previous research findings but also illustrates the potential for structured support programs to facilitate meaningful changes in family dynamics and contribute to children's developmental processes.

In terms of the program's impact on family relationships, key themes such as “parental empowerment,” “shared responsibility,” “developing positive communication with the child,” and “reflection” have been identified. Notably, the support of “parental empowerment” and its positive reflections on children have been highlighted. This suggests that the neurodiversity paradigm, which includes the “acceptance” of neurological differences such as autism and dyslexia (Pellicano and den Houting 2022) can positively impact the well‐being of children. At the same time, adopting the neurodiversity approach indicates that it can also reduce the social stigmatization associated with learning difficulties such as dyslexia and reading difficulties.

The findings of this action research align mostly with previous studies that discuss the experiences of children with reading difficulties and dyslexia, as well as their parents, and typically rely on parent/teacher reports (Alias and Dahlan 2015; Delany 2017; Leitão et al. 2017). However, this study extends the existing findings in the literature by delving deeper through both quantitative and qualitative tools. Many themes that evolved and developed before and after the training program (see Table 3) demonstrated the experiences requiring a holistic approach to be addressed in conjunction with the children's families. This aligns with the frequent emphasis in the literature on the multifaceted and interwoven contextual aspects of having and raising a child with different learning needs (Fernández‐Alcántara et al. 2017; Gould and Dodd 2014; Malouf et al. 2017).

This action research, supported by Bronfenbrenner's (1975) ecological systems theory, emphasizes that the challenges or well‐being reflections of the child and family occur at both the micro and mesosystem levels and are often reflections of one another. The findings, in a sense, highlight the importance of considering the processes that the child and family are involved in as holistic and interrelated structures. This approach, both at the level of ecological systems and through personal empowerment, shows that parents of children can develop transformative and constructive strategies to cope with the many challenges they encounter on this difficult journey.

### 4.1 Limitations and Directions for Future Research

The results of this study are limited to 16 parents of children aged 7–10 with learning difficulties in Ankara, Turkey. This small, homogeneous sample has similar demographic characteristics. The diversity of socio‐cultural and socio‐economic characteristics in parents of children with reading disabilities reasonably affects parental experiences (Graham Ma et al. 2016; Villiger 2021). Prior research demonstrated that parents’ socio‐economic status and socio‐cultural characteristics can exert a considerable influence on their involvement and the academic experiences of their children with learning disabilities (Gerdes et al. 2022; Forteza‐Forteza et al. 2021). It is thus recommended that subsequent research works consider a more diverse sample that incorporates a greater range of socio‐demographic characteristics, thus ensuring the broader applicability of the findings.

Despite the limitations of this study, the findings suggest that parents of children with reading difficulties can develop strategies to cope with the challenges they face and that taking action can lead to growth and transformation in their cognitive structures and behaviors through the impact of personal empowerment. The voices of the participants in this study can effectively provide an understanding of parenting a child with reading difficulties and how it continuously affects family life. Future programs and family involvement models aimed at collaboration in education are crucial in offering various paths and strategies to guide families and teachers in this regard. Family education programs are among the most effective ways to emphasize the importance of participation in education, develop effective partnerships, and especially help children in need of support. This study underscores the importance of positioning families as key stakeholders in the educational process.

## 5 Conclusion

This action research proposes a family education model that could fill existing gaps in the literature regarding providing an integrated process‐based support model for children with reading difficulties and their families. The research findings emphasize the complex, multidimensional nature of difficulties associated with reading disabilities under the umbrella of learning disabilities, highlighting that these issues pose significant challenges not only for the child but also for parents and all family members. The study identifies high levels of stress and various difficulties resulting in significant impacts on the family and all aspects of daily life.

Based on addressing the question “How do parents experience coping with a child's reading difficulties?,” this research initiated and implemented an action plan aimed at developing an Integrated Process‐Based Family Education Program. At the conclusion of this program, the phenomenon of “a long and challenging journey towards parental empowerment” was elucidated.

Findings of thematic analysis at the end of the education program revealed developmental and transformative changes within thematic structures of participants, particularly in categories of “diagnosis process” and “effects of reading difficulties.” Personal empowerment was sought through family education developed as an action plan in this research. The program's holistic perspective, focusing on acceptance, strengths‐based approaches, and a neurodiversity stance against labeling, emerges as an opportunity for families and children to “reflect well‐being onto each other.” These themes underscore the necessity for support programs for children to also consider the parental component. When parents feel understood, respected, and fairly treated in school settings alongside their children, the entire family can benefit from these “reflections of well‐being.”

## Author Contributions

**Hülya Tercan**: conceptualization, data curation, formal analysis, methodology, resources, software, validation, visualization, writing–original draft, writing–review and editing. **Pınar Bayhan**: project administration, supervision, writing–review and editing.

## Ethics Statement

All procedures performed in studies involving human participants were in accordance with the ethical standards of the institutional and/or national research committee and with the 1964 Helsinki Declaration and its later amendments or comparable ethical standards. Hacettepe University Research Ethics Committee granted the relevant approval for our study (Number: 16969557‐903, Decision Number: 2022/08‐26, and Date: February 15, 2022).

## Consent

Written informed consent was obtained from all participants.

## Conflicts of Interest

The authors declare no conflicts of interest.

### Peer Review

The peer review history for this article is available at https://publons.com/publon/10.1002/brb3.70269.

## Data Availability

The datasets generated during and/or analyzed during the current study are available from the corresponding author upon reasonable request.
